# Brain modes of resonance estimated by a biophysical multi-compartment finite elements model

**DOI:** 10.1038/s41598-025-00938-y

**Published:** 2025-09-30

**Authors:** Inês Gonçalves, Dulce Oliveira, Catarina Rocha, Joana Cabral, Marco Parente

**Affiliations:** 1 Department of Mechanical Engineering (DEMec), Faculty of Engineering, University of Porto (FEUP), R. Dr. Roberto Frias, 400, 4200-465 Porto, Portugal; 2 Biomechanic and Health Unity (UBS), Institute of Science and Innovation in Mechanical and Industrial Engineering (INEGI), R. Dr. Roberto Frias, 400, 4200-465 Porto, Portugal; 3 Life and Health Sciences Research Institute (ICVS), University of Minho (UM), Campus of Gualtar, 4710-057 Braga, Portugal; 4 Department of Bioengineering (DBE) and Institute of Systems and Robotics (ISR-Lisboa), LARSyS, Instituto Superior Técnico, University of Lisbon (IST-UL), Av. Rovisco Pais 1, 1049-001 Lisbon, Portugal

**Keywords:** Brain activity, Brain eigenmodes, Natural frequencies, Finite element simulation, Biomedical engineering, Mechanical engineering, Neurological disorders

## Abstract

Neuroimaging studies reveal correlated brain activity across distant regions, suggesting underlying mechanisms that constrain brain function beyond the complex interactions between neurons. Despite these findings, the origins of these patterns and their alterations in neurological disorders remain unclear. Current literature suggests that these patterns could be explained by standing waves resonating within the brain, similar to the vibrational modes observed in musical instruments. Studies have successfully reconstructed brain activity by superimposing resonance modes predicted from the brain’s surface mesh or network structure. However, the role of the brain’s mechanical properties beyond mere geometry and connectivity-such as tissue rigidity and viscosity-, remains largely unexplored. This work aims to fill that gap by demonstrating that the shape of brain modes is also influenced by the physical properties of brain materials, providing a possible mechanistic explanation for alterations observed across cognitive states and mental conditions, when brain shape and connectivity remain unchanged. The brain’s eigenmodes and corresponding eigenfrequencies were analyzed through finite element simulations in Abaqus, incorporating distinct mechanical properties for various brain structures. This study confirms that the brain’s resonance modes are influenced by these properties and highlights similarities between the simulated eigenmodes and fMRI patterns observed in human brains.

## Introduction

The brain, the most complex organ in the human body, processes and interprets all external information, sending and receiving chemical and electrical signals throughout the body. Brain imaging technologies allow neuroscientists to create detailed pictures of brain activity and related them with mental illness, cognition, perception, and emotion^1^. Neuroimaging studies using functional magnetic resonance imaging (fMRI) technology reveal complex patterns of correlated brain activity in both humans and other mammals, which are often disrupted in neurological and psychiatric disorders. Some theoretical models propose that these patterns could be explained by standing waves resonating within the brain’s structure, similar to vibration modes in musical instruments. Supporting this hypothesis, research conducted by Cabral and colleagues at high temporal resolution demonstrated that these correlated fluctuations are synchronous oscillations in signals across distant brain regions in rat brains, suggesting that these resonant waves or modes play a crucial role in brain function^2^.

Despite significant advances, the mechanisms underlying the formation and alteration of these brain activity patterns remain poorly understood. For decades, scientists believed that the key to explaining these structured patterns lay in the complex connectivity between neurons. However, neural field theory suggests that waves of activity propagate across the cortex through the excitation of resonant modes, indicating that brain geometry may impose more fundamental constraints on brain function than long-range neuronal connections between brain regions. Consequently, eigenmodes provide a physical basis for understanding how diverse patterns of correlated brain activity emerge from the brain’s anatomy, shaped by its structural, physical, geometric, and anatomical properties^2,3^. Eigenmodes describe the natural vibration patterns of a system, each consisting of a specific frequency (eigenfrequency) and its associated mode shape (given by its eigenvector), which represents the spatial distribution of displacement. In 2016, Atasoy and colleagues introduced the Connectome-Specific Harmonic Waves (CSHW) framework, providing a frequency-specific representation of cortical activity based on the human brain’s functional networks^4^. This approach derives eigenmodes from the connectome, an undirected and unweighted graph representation of the human brain. In 2023, Pang and colleagues advanced this concept by deriving these representations from the brain’s physical shape, validating neural field theory predictions^3^. However, this approach fails to explain changes in patterns observed in diseases where brain geometry is not altered. Understanding the mechanisms behind long-range brain interactions could revolutionize disease diagnosis and treatment by enabling the prediction of functional activity patterns based on a patient’s brain anatomical and mechanical properties. For instance, if a specific resonant mode is absent in a patient, it may be possible to explore strategies to stimulate or restore that mode. While brain shape strongly influences these modes, the objective is not to alter brain geometry but rather to investigate how modifying mechanical properties-such as tissue rigidity or viscosity of the brain and cerebrospinal fluid-could affect wave propagation. These properties could potentially be modulated through pharmacological interventions or other therapeutic approaches. Since resonance modes depend on factors beyond geometric shape alone, this work is motivated by the hypothesis that changes in brain activity patterns seen in certain disorders may result from alterations in physical properties of the medium through which these waves propagate-factors that are often overlooked when relying solely on surface mesh estimations.

Modal analysis is a crucial engineering technique used to assess the vibration characteristics of structures and materials by identifying their natural frequencies, mode shapes, and damping ratios^5^. While the application of modal analysis to the human head has primarily focused on understanding mechanical impacts and predicting traumatic brain injuries, this research aims to explore the influence of the elastic mechanical properties of brain structures—white matter, gray matter, and cerebrospinal fluid—on the brain’s eigenmodes^5–7^.

Earlier studies on head vibrations relied on experimental tests using animals, cadavers, and volunteers, raising ethical and moral issues and encountering limitations due to a scarcity of subjects and non-standardized procedures. Finite element method (FEM) simulations offer a cost-effective alternative, enabling various analysis without the experimental ethical limitations. Results regarding the natural frequency range of the human head, skull, and brain vary across the literature depending on the methods employed^8^.

The reliability of FEM models of the human head depends strongly on the accuracy of the mechanical properties defined for the different brain components. Most significant differences across brain models stem from the constitutive laws and material parameters chosen to represent brain tissue behavior. Despite efforts to model brain tissue mechanics in both health and disease, contradictory experimental results have impeded progress, largely due to the complexity, softness, and heterogeneity of brain tissue^9–12^.

Most studies characterize brain tissue as non-linear (hyperelastic), time-dependent (viscoelastic), and nearly incompressible with very low stiffness. These studies consistently show that the mechanical response of brain tissue varies across different anatomical structures, with white matter generally stiffer than gray matter. Additionally, brain tissue is considered nearly isotropic from a mechanical point of view^10,12–14^.

Modeling cerebrospinal fluid (CSF) in computational head models also poses technical challenges, with various approaches employed in the literature. In addition to material properties and the constitutive models used, brain anatomical elements and boundary conditions must be carefully selected based on the specific application. Therefore, finite element brain models should be thoughtfully designed to suit their intended purpose^10,15^.

In conclusion, much progress has been made in understanding the brain’s mechanical behavior, the diversity in modeling approaches and parameters highlights the need for further research to improve the accuracy of models and simulations.

## Material and methods

### Brain model

The 3D brain model developed for this study is a heterogeneous brain model that includes white matter (WM), gray matter (GM), cerebrospinal fluid (CSF), and a simplified representation of the skull (excluding the mandible). To create the model, an MNI-152 standard-space T1-weighted average structural template image already pre-segmented into WM, GM, and CSF was used^16^. The segmented data were then converted into STL files and processed in Geomagic Wrap to generate a triangular surface mesh, which was then refined in FEMAP to create the final solid mesh. Among the structures, the CSF was the most challenging to mesh due to its thin regions and the need for model continuity. To address this, only its outer surface was retained, with the interior space defined by the gaps between WM and GM. The final CSF solid mesh was generated from a surface mesh created from the WM and GM solid models, ensuring proper integration within the brain model. For the simplified skull, an offset of the exterior surface of the CSF was applied with a thickness of 5 mm^17^.

**Fig. 1:**
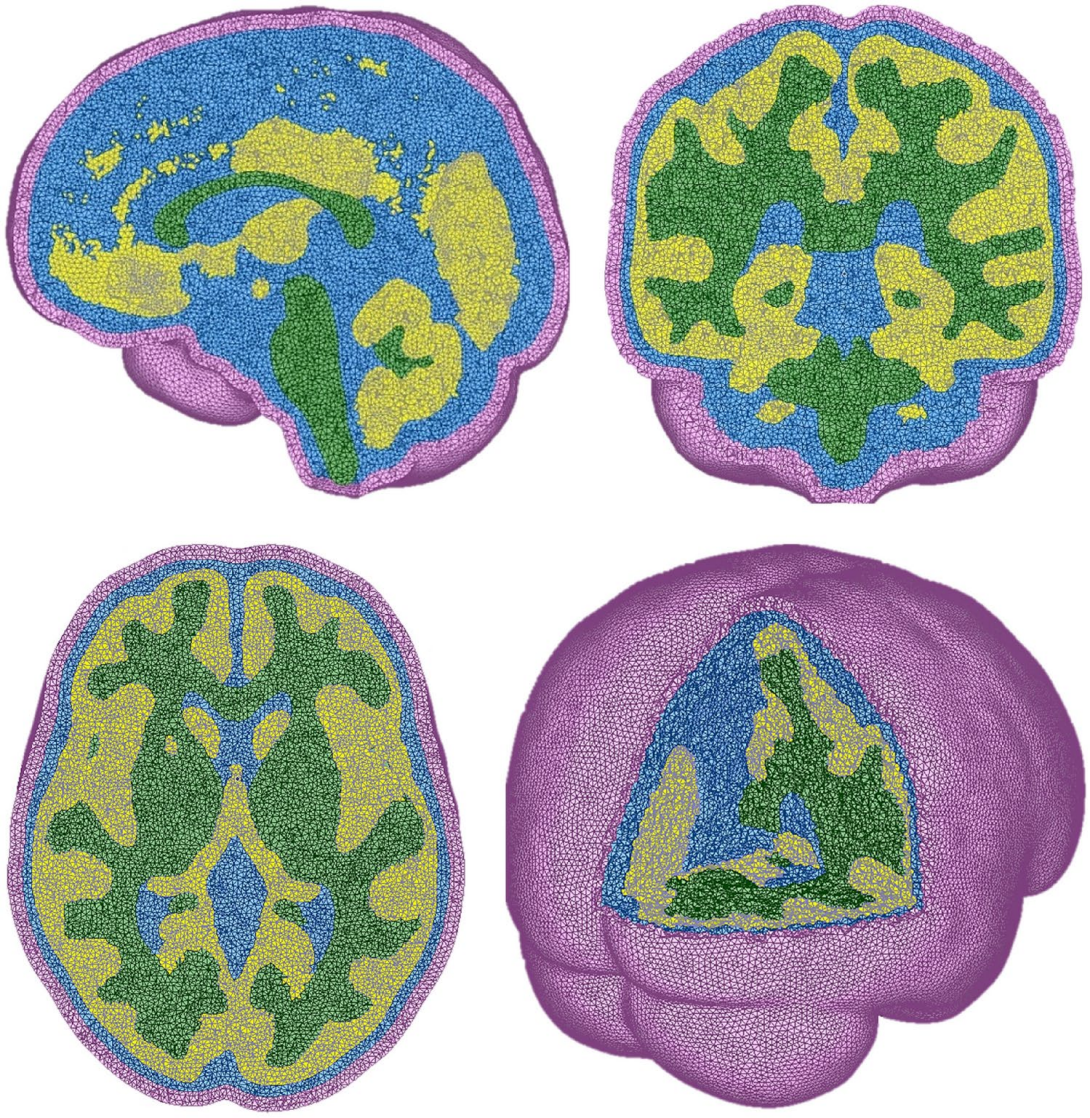
Color-coded representation of the 3D brain model. Pink—Skull, Blue—CSF, Yellow—GM, Green—WM.

Figure 1 shows the final 3D model, which consists of solid tetrahedral elements (C3D4 type). The model comprises a total of 3,638,991 elements and 731,693 nodes. The distribution of mesh elements across the components is as follows: 28.1% in WM, 36.4% in GM, 20.1% in CSF, and 15.4% in the skull.

### Simulation

**Fig. 2:**
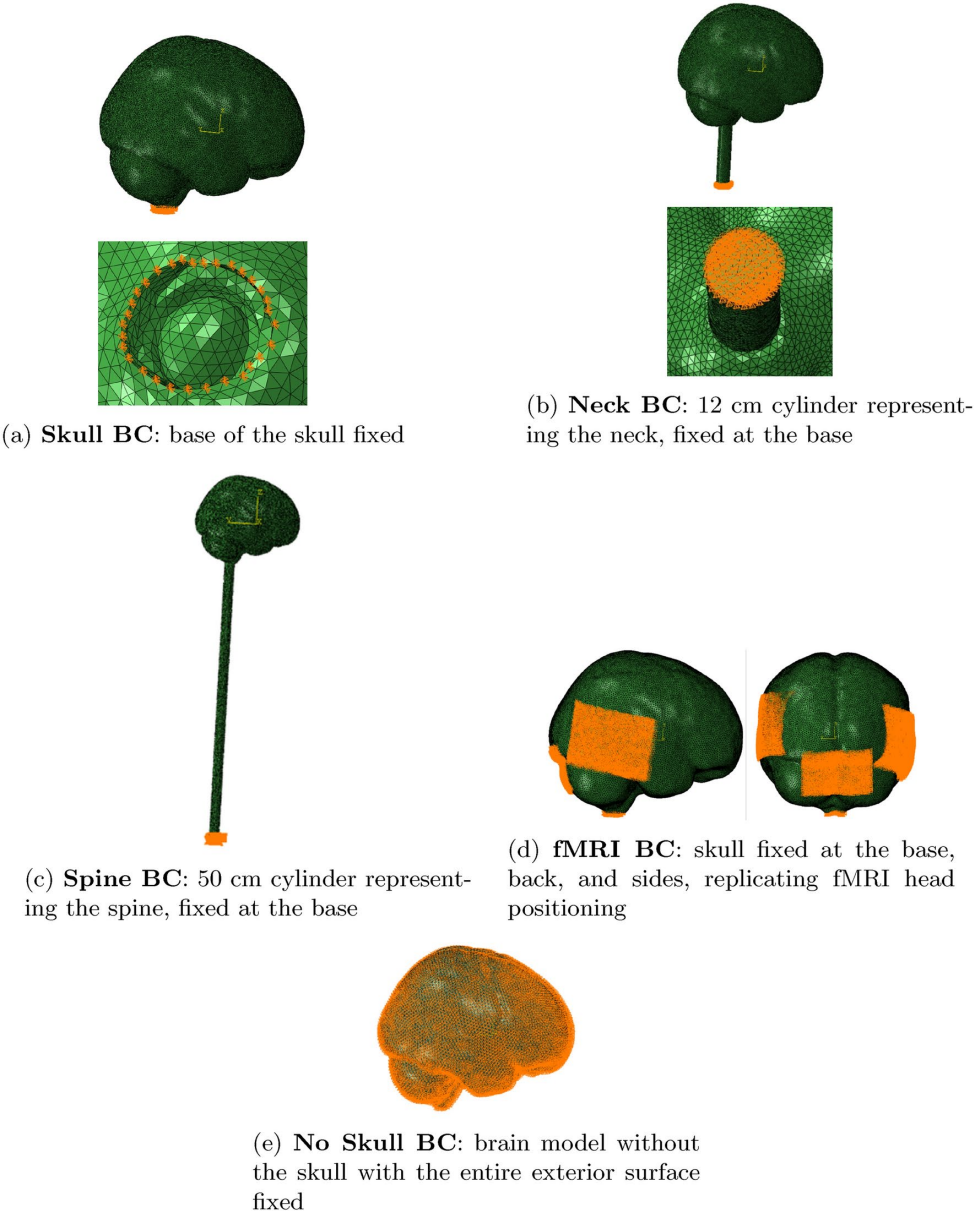
Representation of the boundary conditions defined.

The brain model was simulated using Abaqus software to determine the brain’s natural frequencies and corresponding mode shapes. For that, the boundary conditions, analysis step, and material properties were specified. Several simulations were conducted, each applying a different set of boundary conditions. In one scenario, translational displacements were fixed only at the base of the skull (Fig. 2a). For two other conditions, the neck and spine were simplified as compact cylinders: one with a 12 cm length for the neck (Fig. 2b) and the other with a 50 cm length for the spine (Fig. 2c). In these cases, the base surface of each cylinder was fixed. Another condition involved fixing the skull not only at its base but also at the back and sides to mimicking the immobilization of the head during an fMRI exam, where cushions hold the head in place (Fig. 2d3). Lastly, a boundary condition was applied where the skull was removed, and the entire exterior surface of the brain was fixed (Fig. 2e).

In Abaqus, a frequency linear perturbation step was implemented to extract the brain’s natural frequencies and mode shapes. The governing equation for the dynamic response of the system is given by Eq. 1^8^:

1 $$\begin{aligned} {[}M]\{\ddot{u}\} + [C]\{\dot{u}\} + [K]\{u\} = \{F\} \end{aligned}$$

where [*M*], [*C*], and [*K*] represent the global mass, global damping, and global stiffness matrices, respectively. These matrices are derived based on the chosen element formulation. $\{F\}$ denotes the externally applied load, and $\{\ddot{u}\}$, $\{\dot{u}\}$ and $\{u\}$ are the acceleration, velocity and displacement vectors, respectively, evaluated at the discretized degrees-of-freedom of the system^8^.

Conducting a numerical modal analysis of a system relies essentially on solving the finite element formulation of the free vibration problem (i.e., Eq. 1 with $\{F\}=0$). In the finite element method, the continuous system is discretized in space, leading to the following system of equations for undamped free vibration ($[C]=0$ and $\{F\}=0$)^8,18^:

2 $$\begin{aligned} {[}M]\{\ddot{u}\} + [K]\{u\} = 0 \end{aligned}$$

The displacement vector $\{u\}$ is a function of time and space, which, by assuming harmonic motion for every point of the structure, can be expressed in the form^8,18^:

3 $$\begin{aligned} \{u\} = \{\phi \}_i sin(\omega _it+\theta _i) \end{aligned}$$

where $\{\phi \}$ represents the vector of amplitudes of the degrees-of-freedom, $\omega _i$ is the *i*th natural frequency of the system, *t* is time, and $\theta$ represents the phase angle^19^. Acceleration is the second derivative of displacement for time, being expressed as:

4 $$\begin{aligned} \{\ddot{u}\} = -\omega _i^2 \{\phi \}_i sin(\omega _it+\theta _i) \end{aligned}$$

By substituting the harmonic expressions of $\{u\}$ and $\{\ddot{u}\}$ into the governing equation, Eq. 1 simplifies to:

5 $$\begin{aligned} ([K]-\omega _i^2[M])\{\phi \}_i = \{0\} \end{aligned}$$

The above equation represents a generalized eigenvalue problem, where the goal is to determine both the eigenvalues, $\omega _i^2$, and the eigenvectors, $\{\phi \}_i$. Each eigenvalue corresponds to a unique mode shape vector (eigenvector)^8^. The system as a non-trivial solution, $\{\phi \}_i \ne 0$, if and only if^19^,

6 $$\begin{aligned} \left| [K] - \omega _i^2 [M] \right| = 0 \end{aligned}$$

$\omega _i$ ($=2\pi f_i$) represents the angular frequency of a structure (eigenfrequency in $rads^{-1}$), while $f_i$ denotes the natural frequency (*Hz*). The system is solved by determining each eigenvector and its corresponding eigenvalue using the Lanczos eigensolver for natural frequency extraction^8^, with the process set to extract 25 eigenvalues and apply displacement normalization. The selection of 25 eigenmodes was based on the literature findings, which indicate that lower-frequency modes predominantly govern system behavior^8^. These modes represent large-scale deformations that are more physiologically meaningful, while higher-frequency modes correspond to finer spatial oscillations that may have a lesser impact in this context. This choice ensures that the dominant spatial patterns of interest are well captured within the analysis. During a linear perturbation analysis step, the model’s response is defined by its linear elastic (or viscoelastic) stiffness at the base state. Plasticity and other inelastic effects are ignored^20^. For the material property definition, the mechanical behavior of the brain tissue and cerebrospinal fluid was simplified as linear elastic. Even in this quite restrictive framework, parameter values used to define the mechanical behavior of the various brain components modeled vary widely in the literature^21^.

In the simulations conducted, different material property values were defined, as detailed in Table 1. These values were sourced from experimental studies on brain mechanical properties and finite element (FE) models of the brain found in the literature. For simulations with neck and spine boundary conditions, the material properties of the cylinders representing these structures were identical to those used for the skull. Consequently, this approach simplifies the model by representing the neck and spine solely with bone properties, neglecting the effects of intervertebral discs that facilitate vertebral movement.

**Table 1:** Summary of parameter values used to characterize the material properties of brain structures in the simulations, along with corresponding literature references.

Brain structure	Material property	Value	References
Skull	*E* (MPa)	15000	^5,22^
		8000	^8,23^
		6000	^24,25^
	$\rho$ (g/cm$\phantom{0}^3$)	4.74	^8^
		3.5	^25^
		2.07	^24^
		1.8	^5,22^
	$\nu$ (-)	0.22	^8,19^
CSF	*E* (MPa)	2.19	^26^
		1.314	^8^
		0.299	^5^
		0.1485	^27^
		0.012	^24^
		0.001	^28^
	$\rho$ (g/cm$\phantom{0}^3$)	1.04	^5,19^
		1.00	^23,25^
	$\nu$ (-)	0.4999	^8^
		0.496	^5^
		0.485	^27^
Brain	*E* (MPa)	0.497	^8^
		0.03103 (WM)	^29^
		0.01537 (GM)	^29^
		0.012	^5^
		0.00812 (GM)	^7^
		0.00609 (WM)	^7^
		0.001895 (WM)	^9^
		0.001368 (GM)	^9^
	$\rho$ (g/cm$\phantom{0}^3$)	1.14	^8^
		1.05	^5^
	$\nu$ (-)	0.48	^8^
		0.45	^5^

### Analysis procedure

The results of the simulations, including the mode shapes and natural frequencies of the eigenmodes, were analyzed using multiple approaches. First, to investigate the impact of introducing heterogeneity and including CSF, the results from the heterogeneous brain model were compared with those from a simpler homogeneous model, which represented only the brain without additional structures such as the CSF or skull. This comparison aimed to assess the influence of increased model complexity on the eigenmodes and eigenfrequencies. To ensure consistency, the skull was removed from the heterogeneous model, and the boundary condition applied in both models involved fixing the entire exterior surface of the brain (as in Fig. 2e). This approach eliminated any discrepancies that might arise from differing boundary conditions. The mode shapes were compared qualitatively, without numerical quantification of the similarity between displacement distributions. For the heterogeneous model, WM, GM, and CSF are characterized by their respective elastic moduli (*E*), densities ($\rho$), and Poisson’s ratios ($\nu$). Specifically, the elastic moduli are 0.03103 MPa for WM, 0.01537 MPa for GM, and 0.299 MPa for CSF, with a homogeneous brain model value of 0.02320 MPa. The densities of WM, GM, and CSF are identical, at 1.05 $\times$ 10^−9^ ton/mm$\phantom{0}^3$, while the homogeneous brain model exhibits a slightly different value of 1.04 × 10^−9^ ton/mm^3^. The Poisson’s ratio is 0.45 for WM, GM, and CSF, and nearly incompressible for the homogeneous brain model, with a value of 0.4999.

To assess the impact of varying boundary conditions on the simulation results, the simulations were conducted using a consistent set of material properties across all the boundary conditions previously described. The resulting mode shapes and natural frequencies were then compared to a reference study that performed conventional and advanced modal analyses on a finite element model of the human head and neck system^8^. Since Tse et al.^8^ did not distinguish between WM and GM, the same material properties were assigned to both structures in this work to ensure comparability. Specifically, the elastic modulus was set to 0.497 MPa for both WM and GM, 1.314 MPa for the CSF, and 8000 MPa for the skull. The density values were 1.14 × 10^−9^ ton/mm^3^ for WM and GM, 1.04 × 10^−9^ ton/mm^3^ for CSF, and 4.79 × 10^−9^ ton/mm^3^ for the skull. The Poisson’s ratio was 0.48 for WM and GM, nearly incompressible at 0.4999 for CSF, and 0.22 for the skull. The comparison of mode shapes was conducted qualitatively, without numerical quantification, to assess differences arising from boundary condition variations while maintaining consistency in material properties with the reference study.

**Fig. 3:**
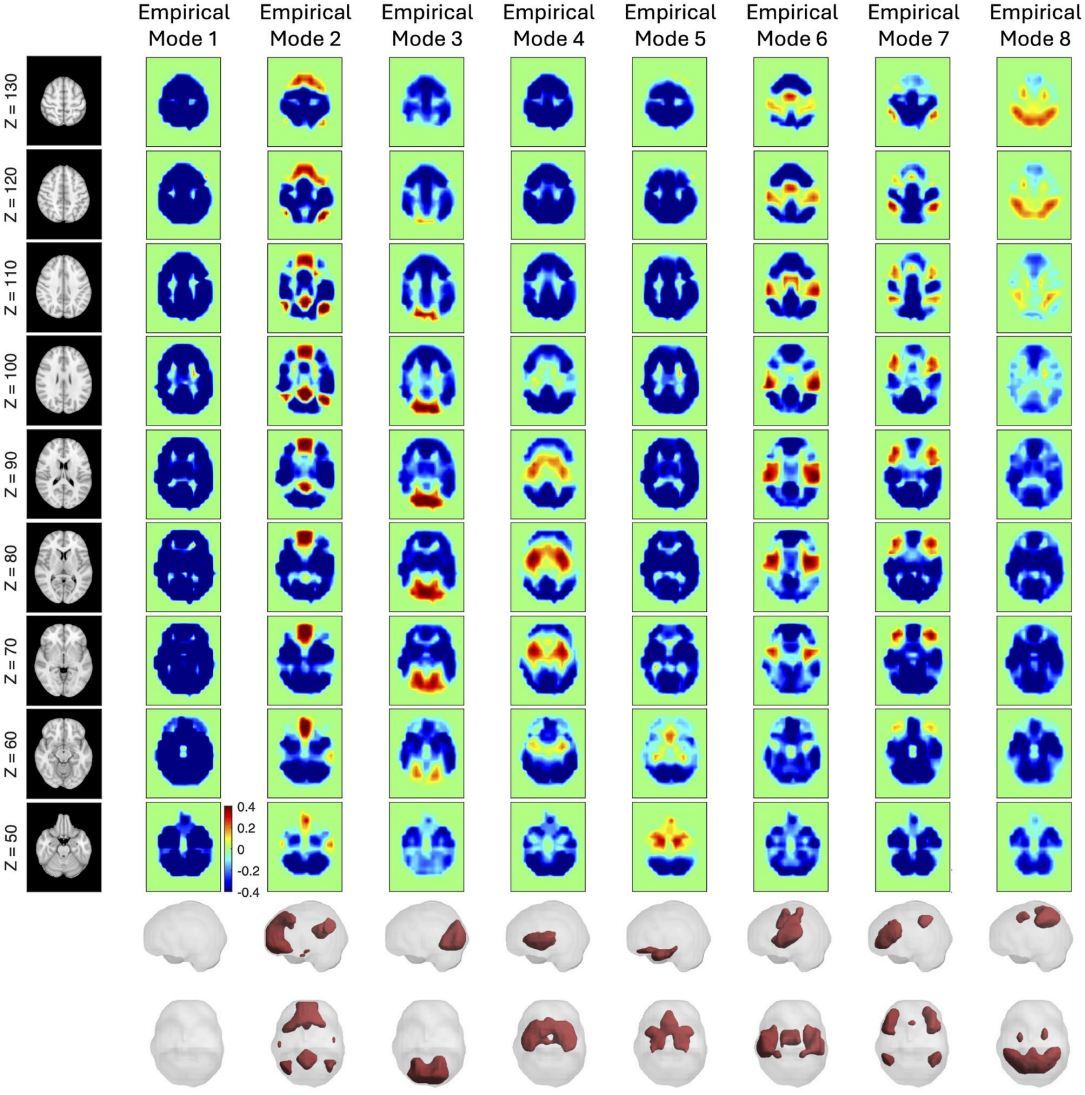
Empirical modes obtained from human resting-state fMRI. Modes were extracted by applying Leading Eigenvector Dynamics Analysis (LEiDA) to 945 preprocessed fMRI scans from the ABIDE public repository. Briefly, the fMRI scans were realigned to a common template, and the patterns of phase alignment obtained at each time point were clustered into K = 8 recurring clusters. The modes represent the K = 8 cluster centroids, each representing a recurring pattern of phase alignment in fMRI signals where the first mode corresponds to a global mode where all signals co-vary in phase, whereas mode 2 the fMRI signals in brain areas associated with the ‘Default Mode Network’ vary in anti-phase with the rest of the brain. The subsequent modes reveal the Visual (3), Ventral Attention (4), Limbic (5), Somatomotor (6), Frontoparietal (7) and Dorsal Attention Networks (8), according to their spatial overlap with intrinsic networks defined in Yeo et al.^32^.

The mode shapes obtained from simulations using various combinations of material properties of brain structures, as reported in the literature, were compared with brain activity patterns identified through fMRI analysis. These brain patterns are usually found at frequencies near 1 Hz, associated with delta waves, which are characteristic of deep, restful sleep^30^. The simulated modes were compared to eight brain activity patterns depicted in Fig. 3. The comparison focused on the displacement distributions of the simulated modes and the colored regions of the brain activity patterns. It was assumed that regions of high brain activity (marked in red) would correspond to areas of higher relative displacement (also red) in the simulated modes. This assumption aligns with findings in the literature, suggesting that modes are most easily excited where the eigenmode amplitude is larger, potentially creating channels of communication between brain regions^31^.

Cerebrospinal fluid diagnostics has emerged as a valuable tool in assessing neurological diseases, as its composition often changes in several conditions, including dementia^33,34^. Given this, it was deemed important to assess how changes in the mode shapes and natural frequencies are influenced by different Young’s modulus values for CSF, as reported in the literature. To investigate this, the stiffness parameter of the CSF was varied while keeping all other material properties constant. Additionally, two different brain tissue stiffness scenarios-representing higher and lower stiffness-were analyzed to assess how brain tissue properties interact with CSF stiffness in shaping the mechanical response. For GM and WM, the elastic modulus values are 0.01537 MPa and 0.03103 MPa, respectively, in the higher stiffness condition, and 0.001389 MPa and 0.001895 MPa in the lower stiffness condition. The skull’s elastic modulus remains constant at 6000 MPa across all scenarios, while the CSF exhibits variable stiffness, with values ranging from 2.19 to 0.001 MPa, including intermediate values of 1.314, 0.299, 0.1485, and 0.012 MPa. The densities are defined as 1.05 × 10^−9^ ton/mm^3^ for GM and WM, 1.00 × 10^−9^ ton/mm^3^ for the CSF, and 3.50 × 10^−9^ ton/mm^3^ for the skull. The Poisson’s ratios are 0.45 for GM and WM, nearly incompressible at 0.4999 for CSF, and 0.22 for the skull.

The natural frequencies and mode shapes for each value of the CSF Young’s modulus were compared with those obtained using a value of 2.19 MPa. To evaluate the differences in eigenfrequencies and mode shapes between models, two criteria were employed: the Normalized Relative Frequency Difference (NRFD) and the Modal Assurance Criterion (MAC)^35^.

The NRFD quantifies the percentage error between corresponding natural frequencies of the reference model and the validation model, as given by:

7 $$\begin{aligned} \text {NRFD}_i = \frac{|f_{r,i} - f_{v,i}|}{f_{r,i}} \end{aligned}$$

where $f_{r,i}$ and $f_{v,i}$ are the natural frequencies of the reference and validation models, respectively. The NRFD values range from 0 to 1, with 0 indicating no difference and 1 indicating a complete discrepancy. Before applying the NRFD, mode shape pairing is required^35^.

The MAC is widely used for quantitative comparison of modal vectors. Originally developed in civil engineering to validate experimental modal vectors against finite element models, the MAC computes a normalized dot product between experimental and analytical modes. Its application has since broadened to various engineering fields. The MAC is highly sensitive to large differences and less sensitive to small differences in mode shapes. It focuses solely on modal shapes, so it is often used alongside frequency comparison methods^36^. In this study, the MAC is used to compare mode shapes between two analytical models.

Considering the eigenvectors for two systems $\varphi _A$ and $\varphi _B$ for mode *i* and mode *k*, the MAC may be written:

8 $$\begin{aligned} \text {MAC}=\frac{(\varphi ^T_{i,A} \varphi _{k,B})^2}{(\varphi _{i,A}^T \varphi _{i,A})(\varphi _{k,B}^T \varphi _{k,B})} \end{aligned}$$

The MAC varies between 1 and 0, with 1 representing perfect consistency and 0 representing no consistency^37^. In this work, MAC values were calculated using the NVH MAC plug-in from Abaqus. Mode shapes were paired based on the MAC matrix values. The MAC and NRFD indicators were then analyzed for corresponding mode shapes, noting any discrepancies in mode numbers.

## Results

### Influence of model complexity

By comparing the mode shapes obtained from the two models, fifteen modes were identified as common to both simulations. In the heterogeneous model, these mode shapes occurred at slightly higher frequencies than in the homogeneous model. This difference is attributed to the higher Young’s modulus assigned to the CSF structure in the heterogeneous model, which introduces additional stiffness. According to the literature on structural modal analysis, an increase in stiffness or a reduction in mass generally leads to higher natural frequencies^38^. In contrast, the ten remaining mode shapes were unique to each model, with no significant similarities identified between them. This suggests that incorporating additional complexities, such as the CSF, significantly affects the resonant modes of the brain. The CSF was meshed based on the thickness obtained from segmentation, and no limitations were encountered in the finite element analysis. This finding highlights the impact of model complexity on the prediction of modal characteristics.

### Comparison between boundary conditions and with literature

Analysis of the results revealed 36 distinct mode shapes among the first 25 eigenmodes from each simulation. Of these, 16 shapes were common across all simulations, displaying substantial similarity in both mode shapes and frequency despite variations in boundary conditions. Three specific mode shapes were identified exclusively in the skull, neck, and spine boundary conditions, but were absent in the no-skull and fMRI conditions. These modes corresponded to the first three mode shapes and, while qualitatively similar across the skull, neck, and spine scenarios, exhibited significant variations in eigenfrequencies. The frequencies progressively decreased as the fixed boundary moved further from the brain. For mode 1, the frequencies dropped from 24.17 Hz under skull conditions to 10.57 Hz under neck conditions and further to 1.78 Hz under spine conditions. Similarly, for mode 2, the frequencies decreased from 33.33 Hz under skull conditions to 11.12 Hz under neck conditions and 1.79 Hz under spine conditions. For mode 3, the frequencies reduced from 83.08 Hz under skull conditions to 23.93 Hz under neck conditions and 9.90 Hz under spine conditions. These reductions correspond to error percentages relative to the skull condition, reaching up to 94.6% for the spine condition, highlighting the impact of boundary proximity on vibrational behavior. The absence of these modes in the no-skull and fMRI conditions is likely due to the restrictive fixation imposed by these boundary conditions, limiting the brain’s ability to vibrate in these specific patterns. This underscores the critical role of boundary conditions in shaping the brain’s dynamic response.

Two additional mode shapes were unique to the spine and neck boundary conditions, which may be attributed to these boundary conditions having the fixed surface positioned further from the brain.

Among the thirty-six identified mode shapes, six were found uniquely with one of the boundary conditions - skull, neck, or spine - and not in others. The analysis was limited to the first 25 eigenmodes, and some higher frequency modes may not appear due to their frequencies exceeding the 25^th^ mode threshold. Notably, the four highest eigenfrequencies modes were only detected under no-skull and fMRI conditions, all exceeding the 25^th^ mode frequencies observed in other simulations.

All modes identified in the no-skull simulation were also present in the fMRI boundary condition simulation, with frequencies differing by less than 0.2%. This suggests that, for the specified material properties, the introduction of the skull did not significantly alter the displacement distribution or eigenfrequencies of the brain. Although increasing stiffness typically raises natural frequencies, the added mass from the skull counteracts this effect, leading to minimal differences in the observed frequencies.

One mode shape was consistently observed in the no-skull, fMRI, and spine boundary conditions, which involve significant brain fixation or a distant fixed surface. The reasons for its absence in the other conditions (brain fixed at the base and neck) are unclear and warrant further investigation, potentially with varying material properties.

The comparison of mode shapes and eigenfrequencies between the current study and the literature^8^ reveals notable differences depending on the boundary conditions. For Mode 1, the frequency reported in the literature is 33.57 Hz, while the current study found frequencies of 24.17 Hz, 10.57 Hz, and 1.78 Hz under skull, neck, and spine boundary conditions, respectively. Mode 2 shows a similar trend, with the literature reporting a frequency of 62.89 Hz, while the skull, neck, and spine boundary conditions yielded frequencies of 33.33 Hz, 11.12 Hz, and 1.78 Hz, respectively. Mode 5, identified only under the spine boundary condition, has a frequency of 20.45 Hz, which is − 90.8% lower than the corresponding value from the literature (221.51 Hz). Similarly, Mode 9 shows a frequency of 256.40 Hz in the literature, whereas the current study finds 129.04 Hz under fMRI boundary condition and 137.42 Hz under the spine condition. Further comparisons include Mode 13, with frequencies of 283.24 Hz in the literature, and 156.58 Hz, 156.57 Hz, 157.37 Hz, and 157.20 Hz under fMRI, skull, neck, and spine conditions, respectively. Finally, for Mode 25, the frequency in the literature is 356.39 Hz, compared to 188.98 Hz, 188.97 Hz, 189.16 Hz, and 189.07 Hz under fMRI, skull, neck, and spine conditions, with a consistent difference of approximately − 47.0%. The simulation with the spine boundary condition yielded the most matching modes. However, for the first two modes, the displacement distributions from the neck and skull boundary conditions closely resemble those in the referenced study, despite no correspondence for modes 5 and 9. The fMRI boundary condition simulation did not match Modes 1, 2, 3, and 5 from the literature. Regarding resonant frequencies, significant discrepancies were observed for the first three modes in the neck and spine boundary conditions, with high relative errors. For other modes, frequency differences were around 50%. The higher natural frequencies reported in the literature may result from model differences, such as the inclusion of additional structures like septal cartilage and teeth in the reference model, and differences in CSF representation. In the current model, CSF is represented in both the subarachnoid space and ventricles, unlike the literature model.

### Comparison between simulated eigenmodes and brain activity patterns

In simulations with varying sets of material properties, a subset of the 25 eigenmodes from each simulation exhibited similarities to the empirical brain modes identified in the fMRI analysis. Table 2 presents 6 simulated modes resembling the corresponding fMRI brain patterns. Since the fMRI images contain signal from brain tissue alone, the skull was hidden in the visualizations of the simulated mode shapes for consistency.

**Table 2:**
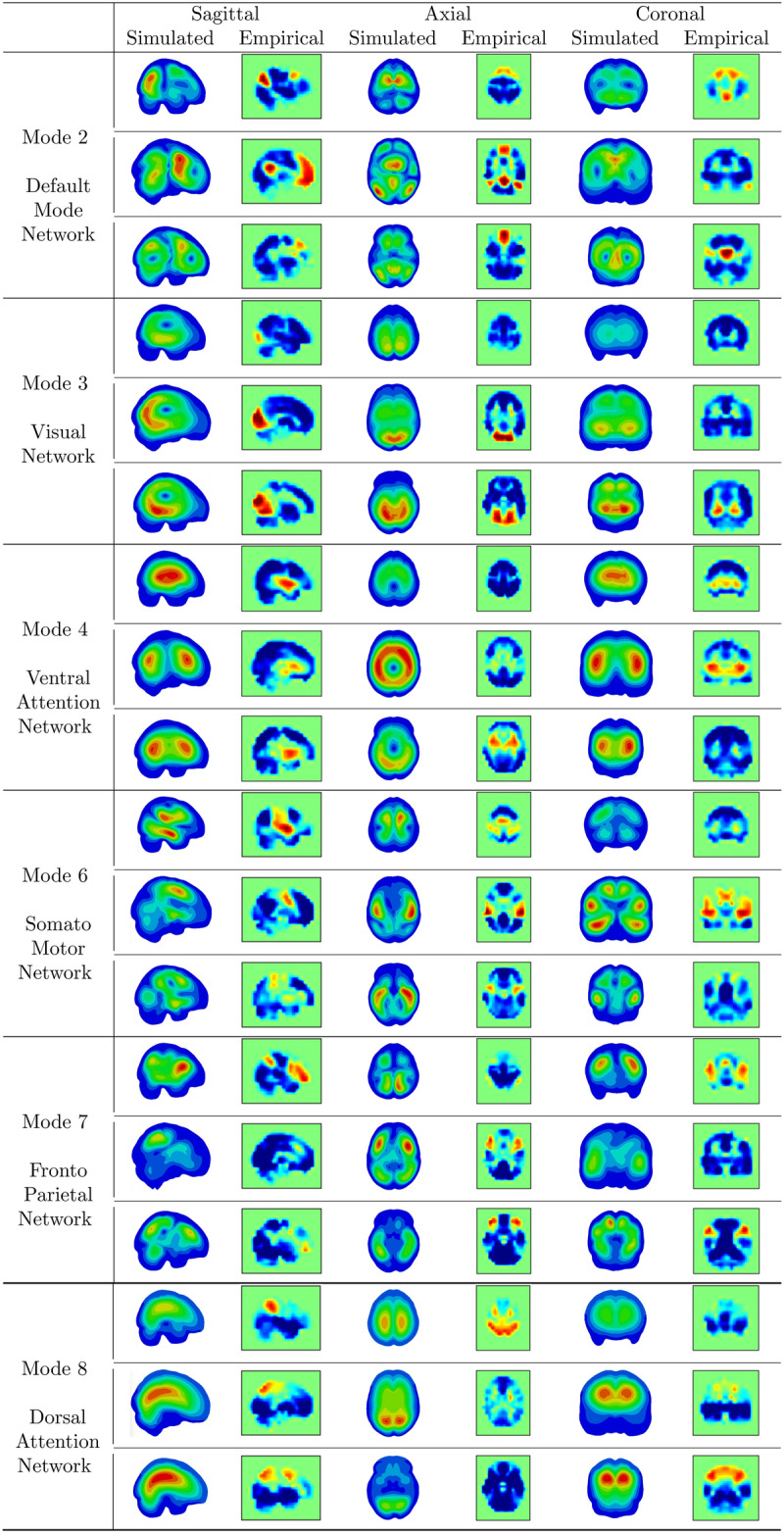
Visual comparison of 6 empirical modes obtained from neuroimaging and 6 modes of resonance obtained from simulations.

No simulated modes were found to correspond to the empirical brain modes 1 (corresponding to globally correlated fluctuations) and 5 (with a pole of displacement in limbic areas). Moreover, although some eigenmodes resembled the fMRI brain patterns, the frequencies at which these modes resonate were significantly outside the frequency ranges observed in the fMRI studies.

### Influence of CSF Young’s modulus

Tables 3 and 4 present the first 10 mode shapes and corresponding natural frequencies for simulations with different CSF Young’s moduli. Table 3 covers simulations with higher brain stiffness (E_GM_ = 0.01537 MPa, E_WM_ = 0.03103 MPa), while Table 4 focuses on lower brain stiffness (E_GM_ = 0.001389 MPa, E_WM_ = 0.001895 MPa).

**Table 3:**
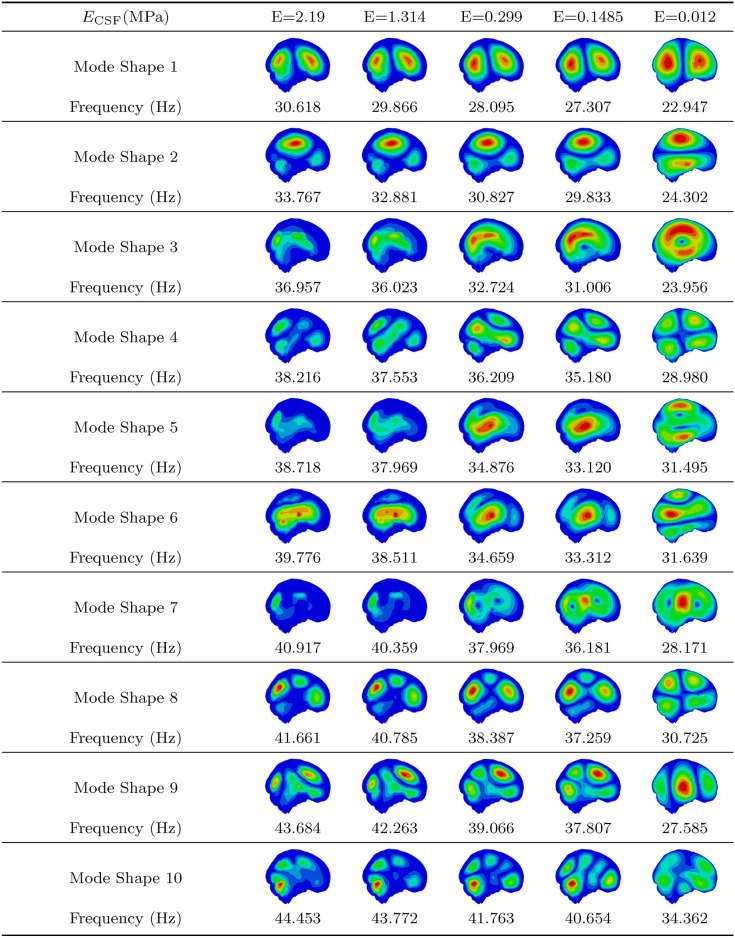
First 10 mode shapes for a higher brain tissue stiffness with $E_{\text {GM}}$ = 0.01537 MPa and $E_{\text {WM}}$ = 0.03103 MPa across varying CSF Young’s modulus values.

**Table 4:**
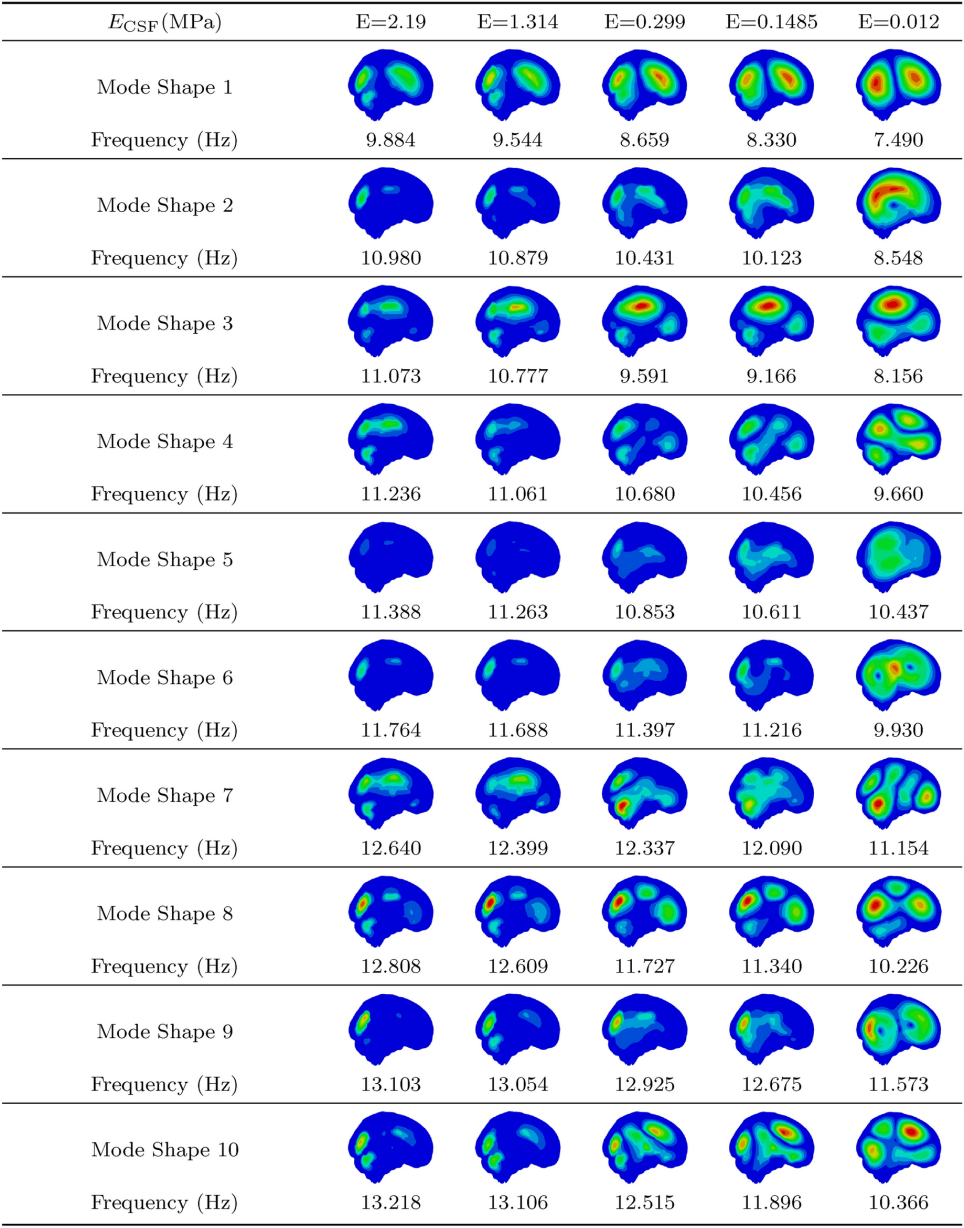
First 10 mode shapes for a lower brain tissue stiffness with $E_{\text {GM}}$ = 0.001389 MPa and $E_{\text {WM}}$ = 0.001895 MPa across varying CSF Young’s modulus values.

As expected, simulations with lower brain stiffness resulted in significantly lower natural frequencies. Additionally, a decrease in CSF stiffness led to lower natural frequencies across all modes. Interestingly, as CSF stiffness decreased, mode shapes became more pronounced, with clearer distinctions in displacement between brain regions. In contrast, lower brain stiffness resulted in less distinct mode shapes for the same CSF stiffness.

**Figure 4:**
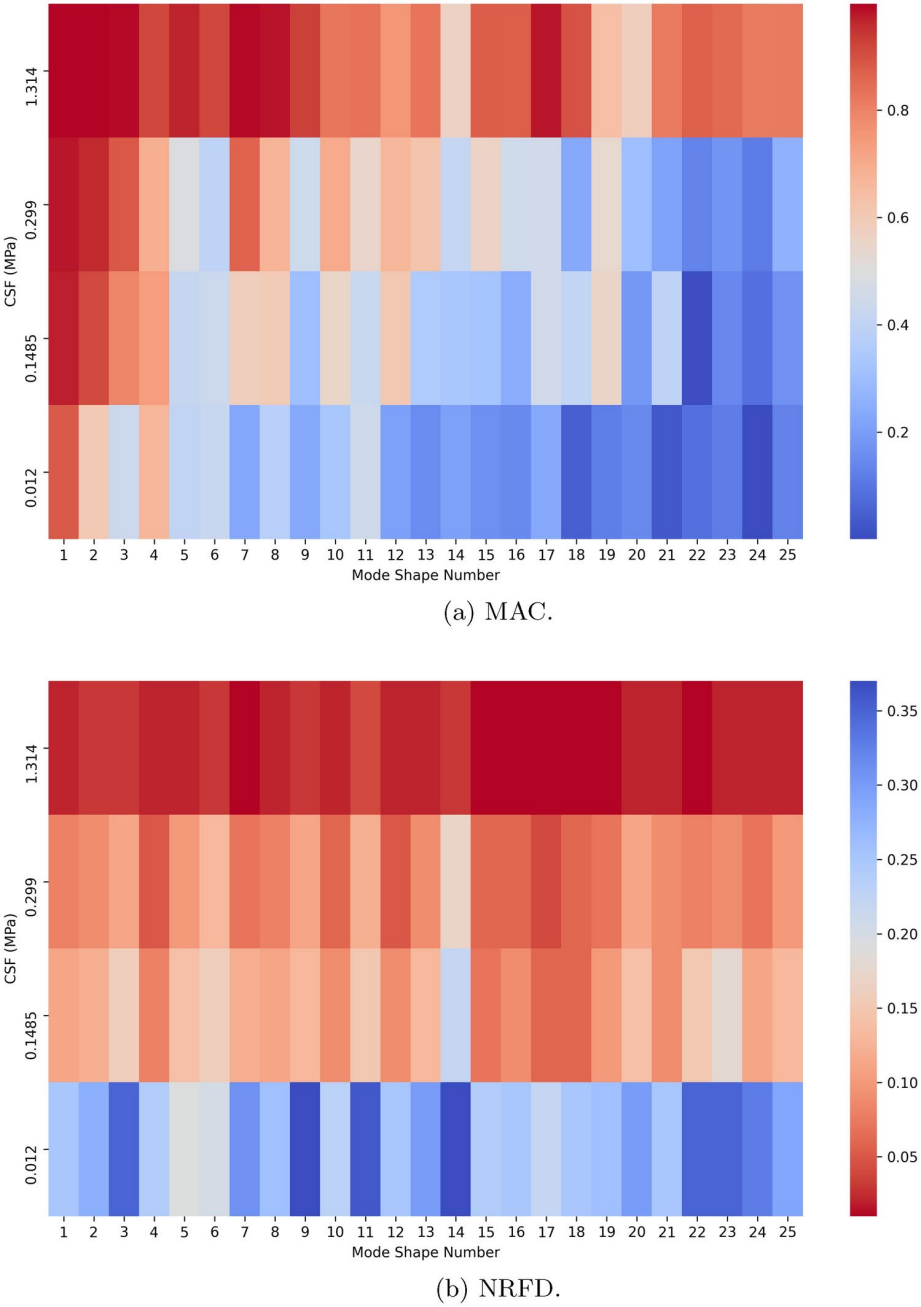
Mode analysis heatmap for higher brain stiffness with $E_{\text {gray matter}}$ = 0.01537 MPa and $E_{\text {white matter}}$ = 0.03103 MPa.

Figure 4 displays MAC and NRFD heatmap for each mode shape identified across simulations, specifically for the case where higher Young’s modulus values were assigned to the brain structures.

**Figure 5:**
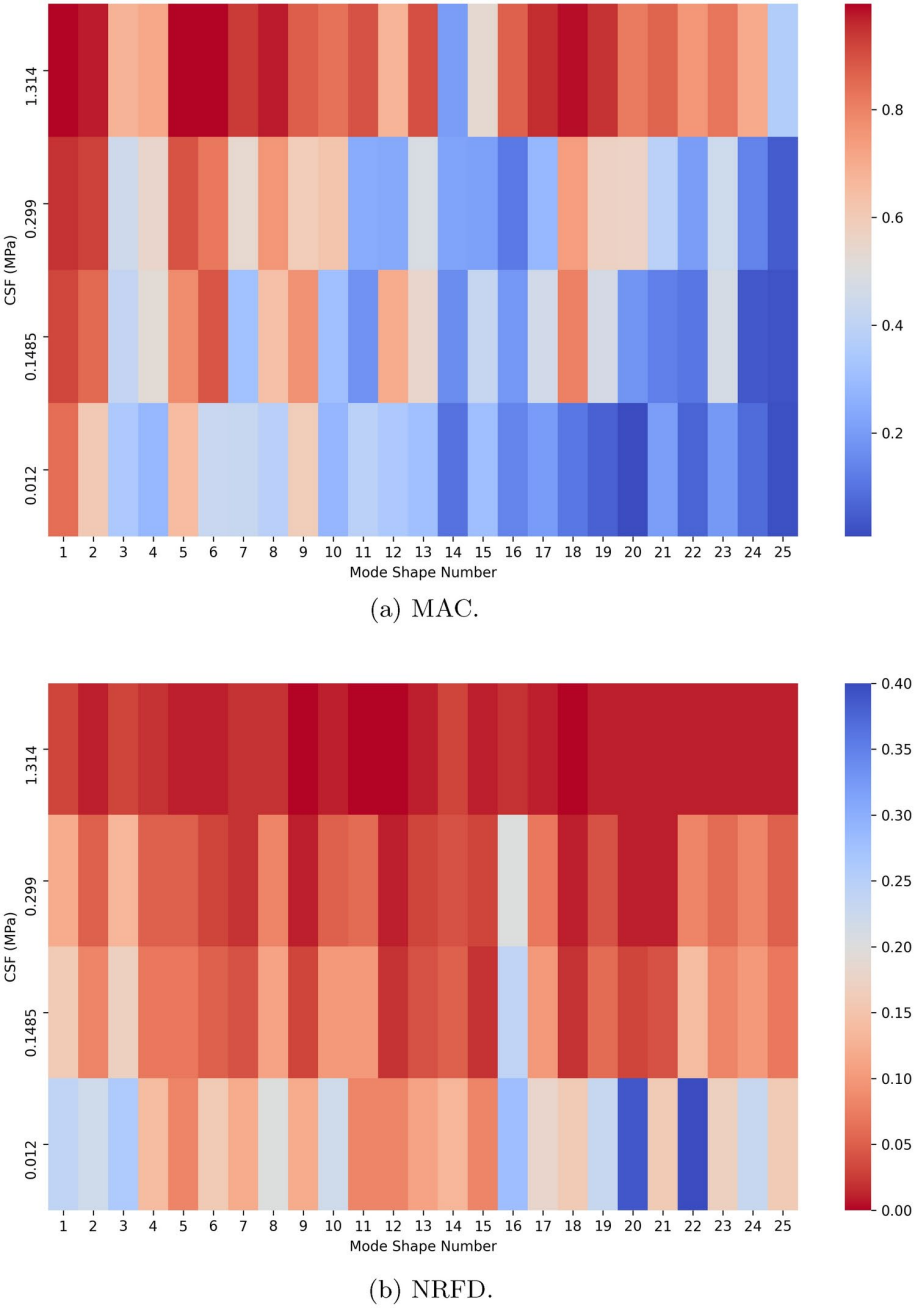
Mode analysis heatmap for lower brain stiffness with $E_{\text {gray matter}}$ = 0.001389 MPa and $E_{\text {white matter}}$ = 0.001895 MPa.

By observing Figure 4a, it is evident that the similarity between mode shapes in the simulations with a Young’s modulus of 2.19 MPa and 1.314 MPa is high, with MAC values exceeding 0.8 for most modes. The lowest similarity is observed for mode shape 14, with a MAC value of 0.57. The frequencies at which the mode shapes occur remain quite similar, with relative frequencies differences below 0.05 (Fig. 4b).

When the CSF stiffness is reduced to 0.299 MPa, the MAC values decrease across all modes, as expected, due to the increased deviation from 2.19 MPa. The NRFD increases alike. In the simulation with a CSF Young’s modulus of 0.1485 MPa, the MAC values are generally lower or comparable to those for the 0.299 MPa case, except for modes 18 and 21, where higher-frequency modes may correlate with higher MAC values beyond the 25th mode. Mode shapes 1–3 maintain high similarity to the 2.19 MPa case, with MAC values above 0.8. The NRFD is comparable between the simulations with CSF Young’s modulus of 0.299 MPa and 0.1485 MPa, though slightly higher in the 0.1485 MPa case. The NRFD increases across all mode shapes compared to the previous simulations.

Reducing the CSF stiffness further to 0.012 MPa results in a general decrease in MAC values for most modes compared to the 0.1485 MPa case. Exceptions include modes 4, 5, 6, and 23, which exhibit similar MAC values for CSF stiffnesses of 0.299 MPa and 0.1485 MPa. This indicates consistent similarity to the 2.19 MPa simulation, though not between these stiffness levels.

Although some modes, such as 3, 8, and 9, display a correlation between the valleys of the MAC chart and the peaks of the NRFD chart (and vice versa), this pattern is not consistent across all modes. For instance, mode 11 shows the opposite trend, making it difficult to draw a clear relationship between the MAC values and NRFD for all modes. However, there is a subtle trend of decreasing MAC values as the mode shape frequencies increase, suggesting that the CSF Young’s modulus has a more pronounced effect on higher-frequency modes.

Figure 5 illustrate the MAC and NRFD for each identified mode shape across the simulations, specifically for the scenario where lower Young’s modulus values were assigned to the brain structures.

In the simulation where the CSF Young’s modulus was set to 1.314 MPa, although the MAC values remained above 0.8 for almost mode shapes, similar to the simulations with higher brain stiffness, two mode shapes (14 and 25) showed significantly lower MAC values, below 0.2 and 0.4 respectively. It is important to note that the mode shape numbers for the simulations with higher and lower brain stiffnesses are not necessarily corresponding mode shapes, as they have not been paired explicitly. Instead, the mode shapes are ordered by increasing frequency from the 2.19 MPa simulation. The frequencies at which the mode shapes occur remain quite similar, with all NRFDs below 0.05, consistent with the results from higher brain stiffness simulations.

When the CSF stiffness was set to 0.299 MPa, the MAC values decreased as expected, and the NRFD generally increased, following the trend observed in previous cases. For the CSF stiffness of 0.1485 MPa, the MAC values were either similar to or lower than those for the 0.299 MPa simulation, with a few exceptions for modes 9, 12, and 15. As before, the NRFD remained similar between these two simulations, with slightly higher values in the 0.1485 MPa case. Mode shapes 1 and 2 retained a high similarity to those from the 2.19 MPa simulation, with MAC values above 0.8.

When the CSF stiffness was reduced further to 0.012 MPa, the MAC values generally decreased across most mode shapes, with some exceptions. Mode shapes 10, 16, 22, and 24 exhibited a similar degree of similarity across the 0.012 MPa, 0.299 MPa, and 0.1485 MPa simulations, all with low MAC values. Conversely, mode shapes 7, 11, 12, 15, and 21 showed higher MAC values in the 0.012 MPa simulation compared to the other two stiffness cases, though none were highly similar to the 2.19 MPa mode shapes. The NRFD increased across most mode shapes compared to previous simulations, with mode shape 11 being a notable exception, though the difference was marginal.

While there is some correspondence between the valleys in the MAC chart and peaks in the NRFD chart, a direct and consistent relationship between the two metrics is not clear. Similar to the higher brain stiffness cases, the MAC values tended to decrease for mode shapes at higher frequencies, especially in the 0.012 MPa simulation.

The minimum natural frequency observed so far was 7.4901 Hz, still significantly higher than the typical brain frequencies measured in fMRI studies. To attempt to achieve values closer to this range, further simulations were conducted with the CSF Young’s modulus reduced to 1 kPa, based on literature on modeling the subarachnoid space^39^. The modes obtained in this simulation (CSF stiffness of 1 kPa) were compared with those from the 12 kPa CSF stiffness simulation. This time, the mode shapes were paired across both CSF stiffness values and different brain stiffnesses. Table 5 presents the first 10 mode shapes, paired between simulations.

**Table 5:**
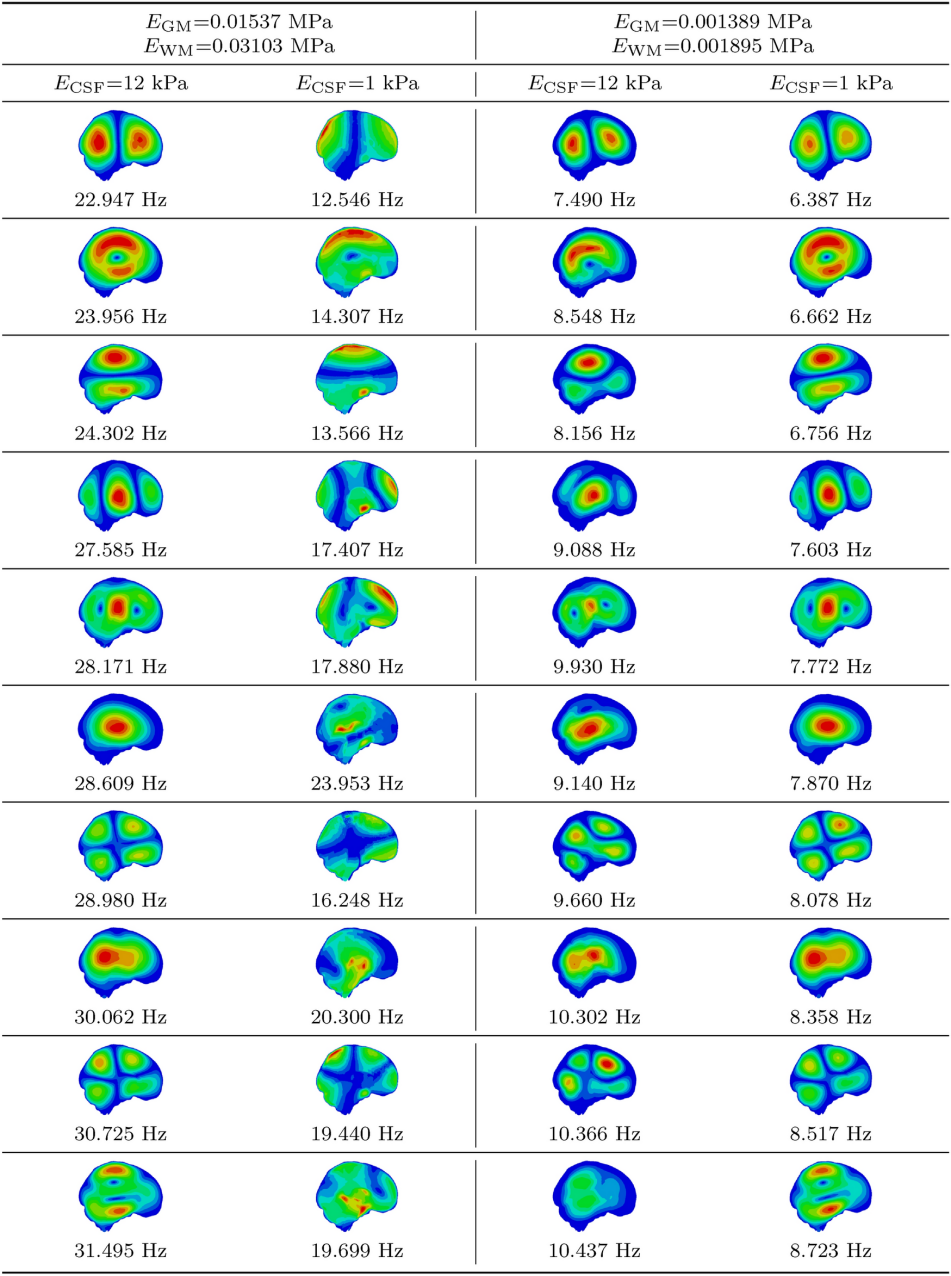
Comparison of the first 10 mode shapes between simulations with a CSF stiffness of 12 kPa and those with a CSF stiffness of 1 kPa.

**Figure 6:**
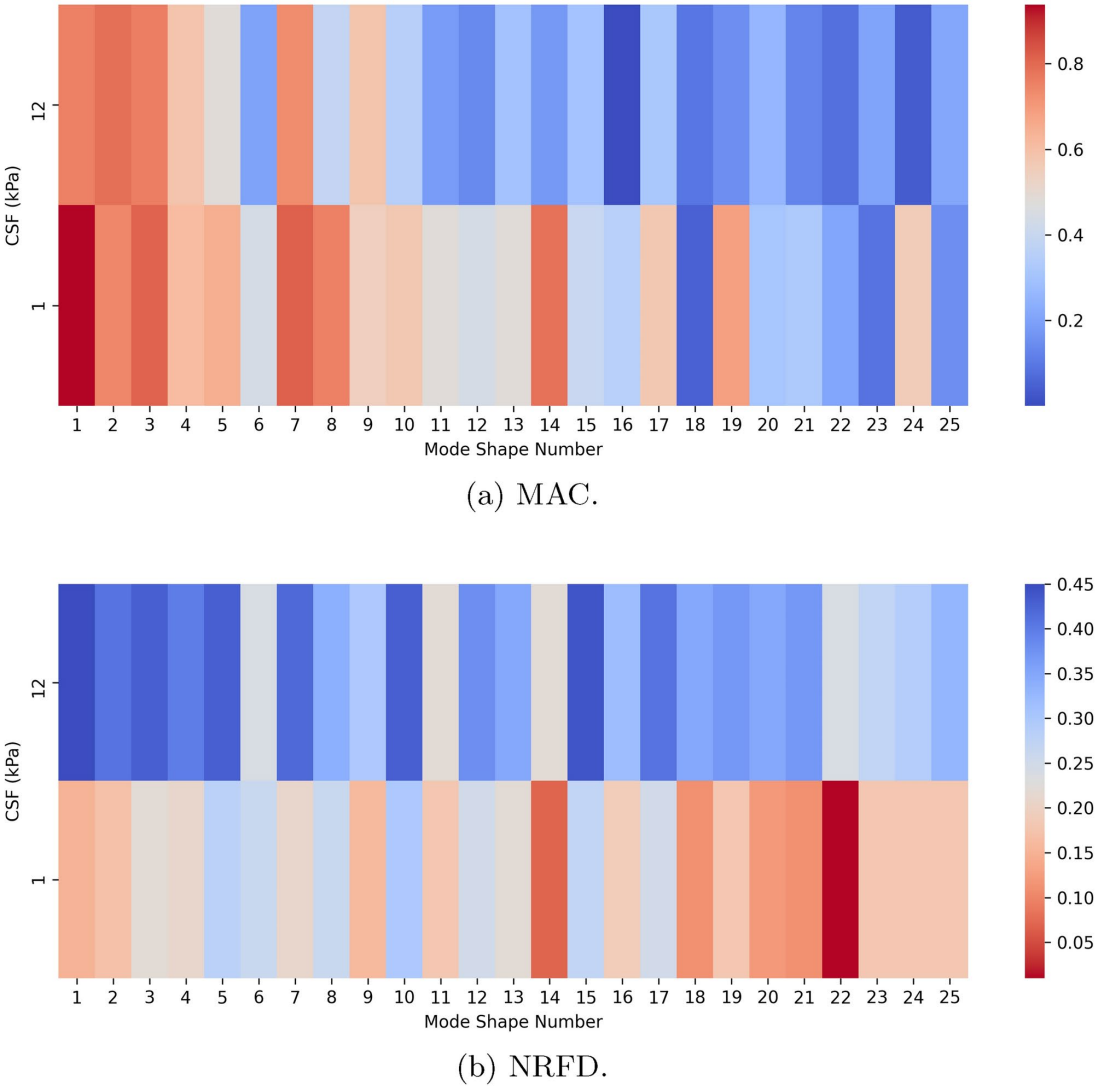
Mode analysis heatmap between CSF stiffness of 12 kPa and 1 kPa.

Figure 6 show the MAC and NRFD between the 12 and 1 kPa CSF stiffness simulations.

The MAC heatmap in Fig. 6a indicates that, in general, the mode shapes exhibited higher similarity in simulations with lower brain stiffness. The NRFD results, observed in Fig. 6b, support this finding, revealing a higher frequency difference when the brain white and gray matter Young’s modulus were defined with higher values. The minimum frequency obtained in the simulation with higher brain stiffness was 12.55 Hz, while the simulation with lower brain stiffness produced a minimum frequency of 6.39 Hz. Although the frequencies decreased, they were still not within the range typically observed in fMRI studies.

These simulations demonstrated a correlation between the stiffness of brain structures and CSF stiffness. To further investigate this relationship, an additional simulation was performed using lower brain stiffness and a CSF Young’s modulus of 0.1 kPa. Although this value is significantly lower than those reported in the literature, it served as a numerical experiment to explore the hypothesis.

Comparison between this simulation and the one with higher brain stiffness and a CSF Young’s modulus of 1 kPa revealed notable similarities in mode shapes: 14 modes exhibited MAC values above 0.8, while the remaining modes fell within the range of 0.5–0.8. These results suggest that the distribution of mode displacements for the resonant modes is more strongly influenced by the ratio of brain to CSF stiffness than by the absolute stiffness values. In contrast, the eigenfrequencies were found to depend directly on the specific Young’s modulus assigned to each structure.

## Discussion

This study investigated several factors influencing the brain’s resonance modes by running multiple simulations. The findings revealed that several aspects of finite element modeling impact the brain’s resonant modes and natural frequencies, particularly the model’s complexity. Specifically, increasing model complexity and incorporating additional structures, such as CSF, were shown to affect the resulting brain resonance modes.

By comparing the results of simulations with different boundary conditions, it became evident that this aspect of finite element analysis also significantly influences the outcomes. While some differences were found in the resonant frequencies when comparing the results with previous literature, the model developed in this work still provided a good approximation. This is supported by the similarity in mode shapes observed between the simulations and the literature^8^. However, the reference study presented only eight of the first 25 mode shapes, which may be an insufficient number for a comprehensive comparison. Additionally, the neck and spine were simplified as compact cylinders neglecting the effects of intervertebral discs, which facilitate vertebral movement. While the cervical spine is a periodic structure that could exhibit bandgap behavior, the primary objective of this study was to capture the dominant modal behavior of the brain and skull, where such periodic effects are expected to have a limited impact on the main eigenmodes^40^. Nevertheless, a more detailed vertebral model incorporating intervertebral discs could provide additional insights into localized wave propagation effects and the role of spinal periodicity in vibrational patterns^41,42^.

These findings reinforce the neurophysiological relevance of the identified eigenmodes, as they align with well-established functional brain networks, including the Default Mode, Visual, Ventral Attention, Limbic, Somatomotor, Frontoparietal, and Dorsal Attention Networks. This correspondence suggests that eigenmode decomposition provides a meaningful representation of large-scale brain dynamics. Furthermore, by extracting these modes from resting-state fMRI data, recurring patterns of phase alignment are captured, offering a dynamic perspective on brain function that complements traditional static connectivity measures. Interestingly, the analysis of simulated resonance modes revealed structural and mechanical factors that may contribute to these observed functional patterns. By comparing the resonant modes with eight functional brain patterns, spatial similarities were identified between regions of high relative displacement in the simulated modes and areas of synchronized activation in functional imaging. Although no exact numerical match was found, this overlap suggests a potential link between mechanical resonance and large-scale brain activity. Moreover, the results indicate that variations in material properties, such as stiffness and density, significantly influence the resulting mode shapes. Different simulations produced distinct brain patterns, reinforcing the idea that brain eigenmodes are not only shaped by neural connectivity but also by intrinsic biomechanical properties. Although this study was based on a single head model, we acknowledge that variations in head shape can affect modal characteristics. In fact, we tested an alternative head model derived from a brain scan of an average-sized brain, though it was modeled as homogeneous. The comparison between the two models highlights differences arising from both geometric variations and mechanical property assumptions. While our findings suggest that head geometry plays a role in shaping modal behavior, further research is needed to isolate its specific effects. A benchmark analysis using an additional head model could provide deeper insights into the sensitivity of eigenmodes to cranial geometry. Future work could explore statistical shape models or parametric variations of head geometry to systematically assess their impact on modal properties.

The study also evaluated the evolution of eigenmodes and eigenfrequencies as the CSF stiffness was reduced, concluding that both the mode shapes and natural frequencies were affected. As expected, a decrease in Young’s modulus led to a reduction in natural frequencies. This analysis demonstrated that the brain’s eigenmodes and natural frequencies depend not only on geometry, as shown in the study by^3^, but also on the medium’s mechanical properties. Interestingly, the influence of altering the CSF Young’s modulus was similar in simulations using both higher and lower brain stiffness values, with few exceptions.

In simulations where the CSF Young’s modulus was set to 1 kPa, observations of the displacement distributions revealed strong similarities between the mode shapes from the higher brain stiffness (with CSF at 12 kPa) and the lower brain stiffness (with CSF at 1 kPa), as shown in Table 5. The calculated MAC matrix indicated that the first 20 mode shapes of one simulation corresponded closely with the other, with MAC values above 0.9. Modes 21–23 had MAC values between 0.7 and 0.8, while modes 24 and 25 had values close to 0, suggesting that their corresponding modes might occur at higher frequencies than those of the 25th mode. Further comparison between simulations with higher brain stiffness and CSF at 148.5 kPa versus lower brain stiffness and CSF at 12 kPa yielded similar results, with displacement distributions showing MAC values above 0.9 for 17 of the 25 modes, and the remaining six modes had MAC values between 0.5 and 0.9.

The comparison between the simulation with a lower brain stiffness and a CSF Young’s modulus of 0.1 kPa and the one with higher brain stiffness and CSF at 1 kPa revealed high similarities in mode shapes, with 14 modes having MAC values above 0.8, and the rest between 0.5 and 0.8. This suggests that the mode displacement distribution of the resonant modes is more influenced by the ratio between brain and CSF stiffness than by the individual stiffness values. However, the eigenfrequencies are directly dependent on the specific Young’s modulus defined for each structure.

Unlike studies that analytically derive modes of resonance from the brain’s surface geometry^3^ or network structure^4^, the work developed here investigates the additional contribution of mechanical eigenmodes of the brain using numerical simulations with a finite element approach, focusing on the influence of the brain’s material properties. These structurally-derived eigenmodes provide insight into how the mechanical properties of the brain influence its vibrational behavior, which may have implications not only for understanding injury mechanisms and mechanical interactions within the brain, but also to shape brain function. While the origin of the functional modes observed empirically in the resting-state remains unclear^2^, structural eigenmodes, inherently associated with the universal principle of resonance, are emerging as a promising candidate explanation, and the method proposed herein to assess these structural eigenmodes offers complementary information about the biomechanical response of brain tissue. The work undertaken in this study contributes to a better understanding of how the brain’s mechanical properties influence its functional activity, although further research is needed in this area. Several limitations and opportunities for future research were identified.

One limitation of this study is the assumption that brain tissue exhibits linear elastic behavior, whereas experimental studies have demonstrated its nonlinear and time-dependent properties. Investigating the effects of viscoelasticity, nonlinearity, and damping on brain eigenmodes could provide valuable insights, as explored by^8^. Additionally, while the brain model included key structures (WM, GM, CSF, and a simplified skull), the CSF was modeled as an elastic solid. Although this approach offers computational efficiency, it does not fully capture the fluid properties of CSF, which influence brain deformation patterns, including shear deformation. The meshing of CSF was particularly challenging due to its thin regions, requiring careful adjustments to maintain continuity and accuracy. Future work should explore fluid-structure interaction methods to better represent CSF dynamics and its biomechanical role. Further validation of the model could involve subject-specific material properties obtained through magnetic resonance elastography, as well as comparisons with experimental data, such as eigenmodes measured in rat brain models. Expanding the model to include additional anatomical structures and more advanced material definitions would further enhance its fidelity and applicability.

Moreover, it is essential to consider more advanced numerical methods, such as design of experiments and machine learning techniques, to better understand the relative effects of the mechanical properties of various parts^43^. These approaches could provide a more comprehensive and quantitative analysis. Moreover, a key step will be to develop a robust method for comparing simulated eigenmodes with brain functional patterns, moving beyond simple visual comparisons of the images. This would allow for more accurate and reliable evaluations of the interactions within the system.

## Data Availability

All data generated or analysed during this study are included in this published article. The MNI-152 standard-space T1-weighted average structural template image, segmented into gray matter (GM), white matter (WM), and cerebrospinal fluid (CSF), is publicly available at https://rdrr.io/github/neuroconductor-releases/MNITemplate/. Brain activity pattern data are available from the corresponding author upon reasonable request.
